# Central Corneal Thickness and Intraocular Pressure in Malay Children

**DOI:** 10.1371/journal.pone.0025208

**Published:** 2011-10-05

**Authors:** Fatemah Heidary, Reza Gharebaghi, Wan Hazabbah Wan Hitam, Nyi Nyi Naing, Nadiah Wan-Arfah, Ismail Shatriah

**Affiliations:** 1 Department of Ophthalmology, School of Medical Sciences, Universiti Sains Malaysia, Kubang Kerian, Kelantan, Malaysia; 2 Unit of Biostatistics and Research Methodology, School of Medical Sciences, Universiti Sains Malaysia, Kubang Kerian, Kelantan, Malaysia; Alcon Research Ltd., United States of America

## Abstract

**Background:**

To determine the mean values for central corneal thickness (CCT) and intraocular pressure (IOP) and the relationship between these values, in healthy Malay children to serve as reference values in diagnosis and treatment.

**Design:**

A cross-sectional study.

**Methodology/Principal Findings:**

One hundred and eight eyes (54 subjects) of Malay children without diagnosis of ocular abnormality or disease meeting our inclusion and exclusion criteria were selected. The CCT and IOP were measured by specular microscopy and non-contact air-puff tonometry respectively, for analysis and comparison with the values obtained in previous studies. Mean CCT and IOP was found to be 530.87±30.79 µm and 15.65±3.05 mm Hg respectively. CCT was found not to vary with age. A positive relationship was found between CCT and IOP; specifically, with every 100-µm increase in CCT, IOP increased by 3.5 mm Hg.

**Conclusions/Significance:**

CCT and IOP are strongly related in healthy Malay children aged 8 to 16. The mean CCT of Malay children is lower than that of majority children of other ethnic groups, supporting the existence of CCT variation among different populations and that ethnicity should be a key consideration when applying CCT data to the general pediatric population.

## Introduction

The measurement of central corneal thickness (CCT) and intraocular pressure (IOP) is essential in the clinical assessment of glaucoma [7]. Recognizing the significance of CCT and IOP in diagnosis, several researchers have investigated their variation among and within various populations [8]. It has been found that low CCT may lead to underestimation of IOP and, as such, potentially impede diagnosis of glaucoma [9]. Based on such findings, it has been suggested that CCT may serve as a surrogate indicator for an abnormal sclera or laminar cribrosa thickness, and possibly as an independent marker for glaucomatous threat [10] .

Researchers have generally established the existence of a positive relationship between CCT and IOP among adults [11], [12], [13], [14], [15]. In children, however, CCT variation among different populations, changes in CCT with age, and the relationship between CCT and IOP remain poorly implicated [9]. Gaining understanding of these variables is essential, as refractive surgery is currently being conducted on an experimental basis in children with anisometropia and bilateral high refractive error [16], [17]. It is particularly important in light of the fact that cornea thickness is a limiting variable in the extent to which refractive errors can be corrected, as only a relatively fixed degree of refractive correction can be performed for each micron of cornea ablated [17]. The mean CCT in “pure” Malay children has not been reported before hence this study aims to determine mean CCT and IOP values for healthy Malay children to serve as reference values in diagnosis and treatment and determine the relationship between these values, if any.

## Materials and Methods

The aim of this cross-sectional, analytical study conducted from January 1 to December 30, 2010 was to develop CTT and IOP profiles of healthy Malay children and determine the relationship between CCT and IOP, if any.

Ethical approval was obtained from the Research and Ethical Committee, School of Medical Sciences, Universiti Sains Malaysia (No.: USMKK/PPP/JEPeM 218.4.2.2). The study was conducted in the Eye Clinic, Hospital Universiti, Sains Malaysia, Kubang Kerian, Kelantan, Malaysia. Written parental informed consent was obtained for all pediatric patients ultimately selected for study inclusion. The study was conducted in accordance with the Declaration of Helsinki.

The study commenced with the collection of demographic data pertaining to age, gender, and ethnicity, the last of which was self-reported by the patient or the patient's parents. Only those potential subjects who self-identified as being of “pure” Malay ethnicity were included in the study. For the purposes of this study, “pure” Malay children were defined as those who descend from at least two generations, which were identified as Malay, spoke Malay as their first language, and practiced Malay customs and Islam [18]. Sample size was calculated using single mean formula [1] with the requirements for level of significance 0.05. Standard deviation (SD) was selected 3.21 [2] and the estimated difference from population mean score was 0.9 giving the sample size 54 respondents. Patients with corneal disease, history of prematurely, intraocular surgery, glaucoma, cataract, eyelid abnormality, IOP greater than 21 mm Hg, or spherical equivalent greater than ±2D were excluded. Patients likely to have abnormally thin corneas, such as those with Down syndrome, Marfan or any other systemic abnormality, were also excluded.

All patients selected for participation underwent assessment of visual acuity with a Snellen chart, slit lamp examination of the anterior and posterior segments of the eye, fundoscopy, air-puff tonometry using a Reichert AT-555 auto noncontact pneumotonometer, and subjective refraction. CCT was measured sequentially on each eye by the same examiner between 8:00 am and 1:00 pm using the Specular Microscope (Topcon Corp., Japan, SP-2000P). One reading (digitalized) was taken. A descriptive analysis was first performed to identify the main trends in the data. Student's t-test was performed to evaluate differences in CCT and IOP between different groups (e.g. boys and girls); one-way ANOVA and Kruskal–Wallis tests were performed to compare CCT among different age groups. Simple linear regression was performed to determine the relationship between two continuous variables (e.g., between refraction and CCT or between refraction and IOP). The data were analyzed with Statistical Package for Social Science (SPSS Inc., Chicago, IL) software version 18.0 at a significance threshold of 5% (*P*<0.05).

## Results

Of the 54 subjects (108 eyes) examined, 28 were boys (51.90%) and 26 girls (48.10%). The mean age of the all subjects (range, 8–16 years) was 12.27±2.76 years. The mean age was 12.35±2.711 years and 12.19±2.87 years for male and female subjects respectively. When the subjects were stratified into 1 of 6 age groups—aged 8 to 9, 10 to 11, 12 to 13, 14 to 15, or 16—the most representative age group was found to be the 14–15 years age group, which accounted for 22.2% (*n* = 12) of the sample.

**Figure 1:**
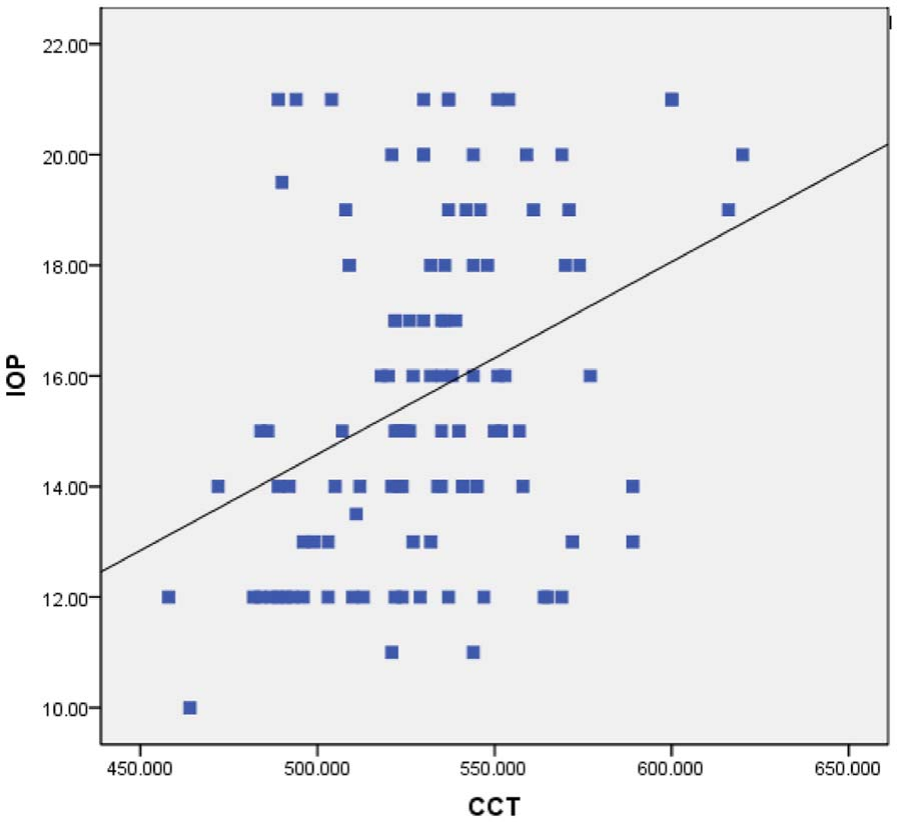
Scattergram of central corneal thickness (CCT) versus intraocular pressure (IOP) of children aged 8 to 16 years (r-sq = 0.12, n = 108).

Tables 1 report the CCT and IOP values obtained by age and gender. As can be observed, the mean IOP was found to be 15.65±3.05 mm Hg and the average CCT was found to be 530.87±30.79 µm. Figures 1 show the relationship between IOP and CCT. As can be observed in the plotting of IOP against CCT according to the results of linear regression analysis yields a line with a nonzero slope (95% CI 0.02, 0.05; p<0.001) with a slope value of a 3.5 mm Hg increase in IOP for every 100-µm increase in CCT. The results of further analysis yielded no significant findings regarding IOP and CCT distribution or refractive errors among the different age groups (*P*>0.05), indicating the existence of no relationship between CCT and IOP by age, the existence of refractive errors or gender (Table 2).

**Table 1:** Mean central corneal thickness (CCT) and intraocular pressure (IOP) according to age and gender.

Age	Mean CCT		Mean IOP	
	Boys	Girls	Boys	Girls
8–9 years	549.30±41.04	510.42±21.46	17.00±3.00	14.00±3.00
10–11 years	535.33±26.44	538.20±26.56	17.00±4.00	16.00±2.00
12–13 years	532.67±10.77	507.63±36.58	14.00±2.00	15.00±4.00
14–15 years	532.70±33.83	521.92±18.88	14.00±2.00	16.00±3.00
16 year	546.00±40.63	534.20±30.32	17.00±3.00	16.00±4.00

Mean ± SD, IOP: Intraocular pressure, CCT: Central Corneal Thickness.

**Table 2:** Comparison of central corneal thickness (CCT) and intraocular pressure (IOP) between boys and girls.

	Parameters	Mean differences (95% CI)	t-stat (df)	p-value^1^
Right Eye				
	IOP	0.48 (−1.13, 2.10)	0.60 (52)	0.550
	CCT	−15.50 (−32.08, 1.08)	−1.88 (52)	0.066
Left Eye				
	IOP	−0.72 (−2.46, 1.02)	−0.83 (52)	0.411
	CCT	−16.60 (−32.97, −0.23)	−2.04 (52)	0.047

1 Student t-test was applied.

## Discussion

**Figure 2:**
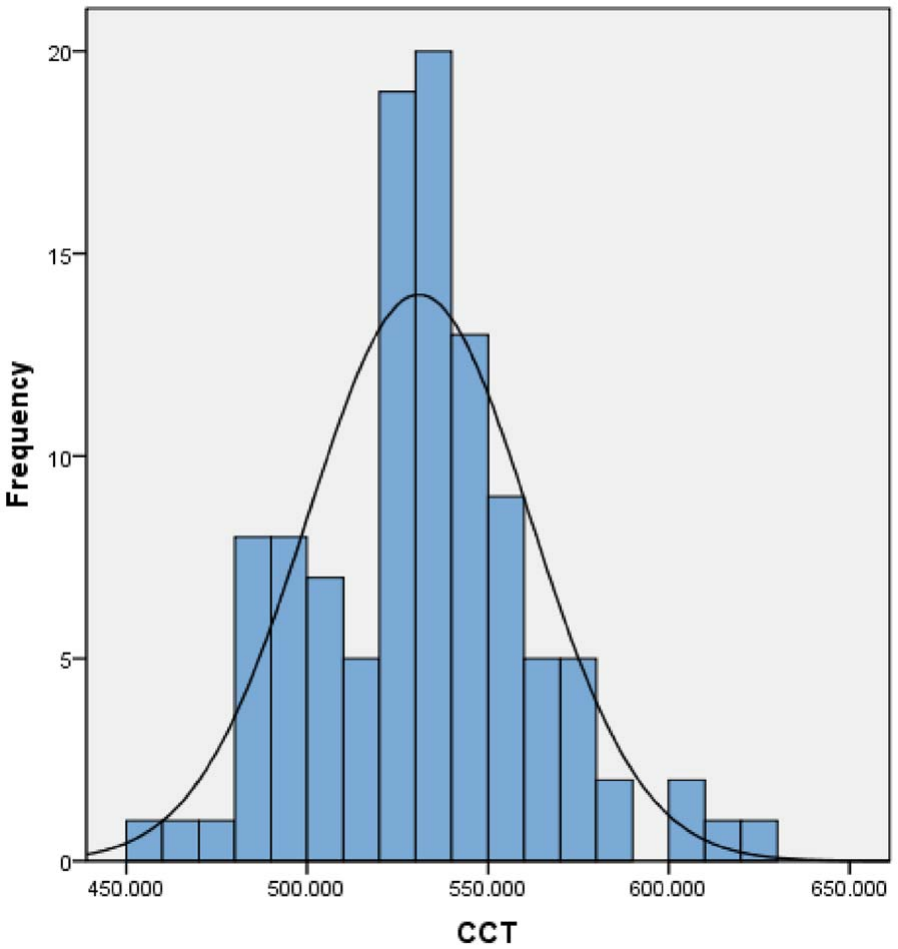
Distribution of central corneal thickness (CCT) in 108 eyes of children aged 8 to 16 years. CCT is normally distributed. The average CCT was 530.87 +/−30.79 µm.

In this cross-sectional, hospital based study of healthy children in Malaysia, we determined the mean CCT and IOP among different age groups of children self-identifying as being of Malay ethnicity and identified a significant relationship between the variables of IOP and CCT. Specifically, we determined the mean CCT of 530.87±30.79 µm with normal distribution (Figure 2), values that are approximately lower than those reported in the majority of previous studies of other races (Table 3). This findingis of particular importance in light of the fact that examination of patients with low CCT may yield erroneously low IOP measurements, which can lead to delay in diagnosis of glaucoma (Table 3). Although a range of genetic and environmental factors appears to contribute to CCT variation [19], the specific variables most responsible for variation have not yet been identified, calling for further research.

**Table 3:** Mean central corneal thickness (CCT) and intraocular pressure (IOP) values obtained in previous studies, a literature analysis.

Study	Age Group	Ethnicity/Race	CCT Value(s) (µm)	CCT Instrument(s)	IOP Value(s) (mm Hg)	IOP Instrument(s)
Haider et al. [9]	7 months–18 years	African-American	535±35	Ultrasound pachymeter	16±4	Tono-Pen
		White	559±38		15±4	
[17]Hussein et al.	6 months–14 years	Caucasian	551±48	Ultrasound pachymeter	N/A	N/A
		Hispanic	550±34			
		African-American	532±48			
[20]Hikoya et al.	8 months–18 years	Japanese	36.9±544.3	Ultrasound pachymeter	13.9±2.4	Tono-Pen
Muir et al. [24]	5–17 years	Black	537±36	Ultrasound pachymeter	19.3±6.0	Goldmann applanation and Tono-Pen
		White	564±28		17.7±4.2	
[26] Yildirim et al.	Mean 10.1±1.6	Turkish	564.92±32	Ultrasound pachymeter	16.7±2	Noncontact tonometer
					17.9±2	Tono-Pen
Muir et al. [2]	9 months–17 years	White	562±35	Ultrasound pachymeter	A/N	A/N
		Black	543±37			
[27]Sahin et al.	7–12 years	Turkish	561.37±33	Ultrasound pachymeter	17.47±2.7	Tono-Pen
					16.81±3.1	Rebound tonometer
Muller et al .[36]	5 to 11 years	White	529±36	Ultrasound pachymeter	15.4±2.4	Non-contact tonometer
[29]Tong et al.	9–11 years	Chinese	546.0±31.8	Non-contact optical pachymeter	A/N	A/N
		Non-Chinese (Malay and Indian)	536.6±31.5			
Dai et al. [30]	1–18 years	African-American	523±40	Ultrasound pachymeter	A/N	A/N
		Caucasian	563±36			
		Hispanic	568±44			
Osmera et al.[31]	7–17 years	Czech	554±33	Ultrasound pachymeter	14.5±2.6	Goldman applanation tonometry
Herse et al. [32]	5–20 years	New Zealand	540±25	Optical pachometry	N/A	N/A
Coste et al. [34]	3 to 16 years	White European Caucasian + North African	529.30±32.53	Specular microscopy	N/A	N/A
Lee et al. [33]	9–11 years	Chinese	N/A	N/A	16.6±2.7	Non-contact tonometry
Doughty et al. [35]	5 to 15 years	White	±529 34	Ultrasound pachymeter and Specular microscopy	16.8±2.9	Non-contact tonometry
Lim et al. [37]	Mean 13.97±0.90 years	Singaporean(Chinese, Malays and Indians)	578.76±34.47	Ocular Response Analyzer	15.12±2.84	Ocular Response Analyzer

N/A: Data not available.

We found the mean IOP of our subjects to be 15.65±3.05 mm Hg. Comparison of mean IOP among different populations reveals that the mean value is greater than Japanese, Singaporean and Czech children, but lower than African American, black, Chinese and Turkish children (Table 3). Such differences in mean IOP and CCT among these groups supports the hypothesis of the existence of structural variations among different ethnic and racial groups [9].

Individuals with lower socioeconomic status are more likely to undertaking numerous diseases [3]. ‘Social determinants in ocular diseases’ is a novel approach to determinate the role of socioeconomic factors in ocular disease [4]. Moreover, It has been shown that subjects with lower income and education have a higher mean IOP [5]. On the other hand, subjects from rural areas have markedly thinner corneas as compare as other races [6]. These findings add to the body of evidence that socioeconomic factors may influence on CCT and IOP. Unfortunately, we were unable to assess socioeconomic status in our subjects. This idea may be consider as a good proposal in performing future studies.

Previous studies have found that different instruments yield different CCT values for the same subject [20], [21]. Specifically, Bovelle et al. reported that measurement by specular microscopy yields significantly lower values than does measurement by ultrasound pachymeter [22]. When Suzuki et al. compared CCT values obtained using Orbscan scanning-slit corneal topography pachymetry, the Topcon SP-2000P, noncontact specular microscopy, and ultrasonic pachymetry within a population, they found that mean CCT values did not significantly differ between those obtained using scanning-slit topography and those using ultrasonic pachymetry. However, they found that mean CCT values obtained by contact specular microscopy were considerably lower than those obtained using the other devices [23].

In this study, we measured ocular parameters using noncontact methods that differed from those used in previous studies. Specifically, we used specular microscopy to measure CCT, while pachymetry had been used in other studies, and performed tonometry by airpuff tonometer to measure IOP, while most other pediatric studies had used the Tono-Pen. As non-contact methods, air-puff tonometer and specular microscopy offer the advantages of causing less discomfort for children and reducing the risk of disease transmission through infected instruments. However, the differences between the measurements obtained using contact and non-contact methods limits their direct comparison, whether within the same study or among related studies.

The relationship between CCT and age remains incompletely understood. Several investigations have identified a negative relationship between CCT and age among adults [9]. In children, CCT has been reported to decrease rapidly during the neonatal period before gradually increasing until the maximum (adult) level is reached at 3 or 5 years of age [20]. Muir et al. suggested that CCT slowly increases in children until 5 years of age, at which point it remains stable before beginning to decrease between 10 to 14 years [24]. Hussein et al. also reported that CCT increases in children until 9 years of age before decreasing between 10–14 years [17]. In a recent study of the relationship between IOP and CCT among children 0 to 10 years of age, Sauera et al. did not find any significant difference in CCT among the different age groups [25]. Likewise, we found no significant difference in CCT, as well as IOP, among the different age groups in our study.

Our findings indicate that for every 100-µm increase in CCT, IOP increases by 3.5 mm Hg. Other studies into the relationship between CCT and IOP have reported a relationship between IOP and CCT in the pediatric population that approaches a level of significance. In a study of Turkish children, Yildirim et al. found that IOP increased 2.1 and 4.2 mm Hg with every 100-µm increase in CCT when using the Tono-Pen and the non-contact tonometer, respectively [26]. In another study on Turkish children, Sahin et al. found that IOP increased 2.3 and 3.5 mm Hg for every 100-µm increase in CCT when using the Tono-Pen and rebound tonometer, respectively [27]. Likewise, Muir et al. identified a relationship between CCT and IOP (*P* = 0.0002), specifically that IOP increased by 2.2±0.6 mm Hg for every 100-µm increase in CCT. [2] Conversely, Haider et al. found no statistically significant relationship between mean CCT and IOP among either White (*R* = 0.18) or African American (*R* = 0.24) children [9].

The major limitations of this study were its cross-sectional nature and the small number of children that we examined in each diagnostic group. Hence, the absence of a finding especially in relation between IOP and CCT with age or gender does not mean that there is not one. As our findings regarding the relationship between CCT and IOP were not based on analysis of longitudinal data, we could not use them to make inferences regarding changes in the nature of this relationship for the individual subject. Despite these limitations, we believe that the strengths of this study, which include examination of a homogenous population representing several different age groups, and use of non-contact means of measurement, make its findings particularly significant.

Recognizing that members of the same ethnic group living in different countries or different regions of the same country engage in different behaviors and are exposed to different environmental variables, we recommend that future research examine Malay children living in other East Asian countries or other states in Malaysia. As we limited our study to children with healthy corneas, additional research is also needed to illuminate the interesting relationship between CCT and IOP in children with unhealthy corneas, a history of pseudophakic eye(s), or a family history of glaucoma. Such research, especially longitudinal studies following the same subjects into adulthood, would also refine our understanding of ocular growth in terms of biometric changes of the cornea, as well as the relationship among other ocular parameters, such as axial length, corneal curvature, and endothelial cell density.

Knowledge of normal ocular structures in different races would offer a significant reference value and may assist in the identification of various diseases including glaucoma [28]. Given the increasing importance of CCT knowledge and appropriate measurement in diverse areas ranging from glaucoma diagnosis to refractive surgery, we argue that patient ethnicity should be key considerations when applying clinical data regarding factors known to be influenced by CCT and IOP to the general pediatric population. Our argument is reinforced by our finding of a lower mean CCT in the Malay pediatric population compared to other populations, which provides evidence of the existence of CCT variation among different populations.
