# Correction: Central Corneal Thickness and Intraocular Pressure in Malay Children

**DOI:** 10.1371/annotation/627b0a20-6623-4d46-970a-2f1f2ad0d002

**Published:** 2011-12-27

**Authors:** Fatemeh Heidary, Reza Gharebaghi, Wan Hazabbah Wan Hitam, Nyi Nyi Naing, Nadiah Wan-Arfah, Ismail Shatriah

There was an error in the first author's given name. The correct name is Fatemeh Heidary. 

